# Unmet community care needs and older adults’ well-being: the moderating role of childlessness in China

**DOI:** 10.1186/s12877-025-06952-z

**Published:** 2026-02-04

**Authors:** Shibin Yan

**Affiliations:** Department of Public Policy and Administration, Rutgers University, 401 Cooper Street, Camden, NJ 08102 USA

**Keywords:** Community care services, Unmet needs, SWB, Health status, Childlessness, Older adults, China

## Abstract

**Background:**

As China’s population ages rapidly amid declining traditional family support and rural–urban migration, community care services have emerged as a vital solution for eldercare. However, disparities between service demand and provision have created widespread unmet needs. This study investigates how unmet community care needs impact the health status and subjective well-being (SWB) of older adults, with attention to the moderator role of childlessness and variations across service types.

**Methods:**

The study analyzed four waves of data from the Chinese Longitudinal Healthy Longevity Survey (CLHLS) from 2008 to 2018, using two-way fixed-effects regression models (*N* = 8,301). Health status was assessed using self-rated, interviewer-rated, and comparative health, while SWB was measured by life satisfaction, positive affect, and negative affect.

**Results:**

Unmet needs were associated with lower life satisfaction (β = -0.010, *p* < 0.01), reduced positive affect (β = -0.024, *p* < 0.01), and increased negative affect (β = 0.023, *p* < 0.01). Unmet needs also reduced self-rated health (β = -0.005, *p* < 0.05). These effects were weaker among childless older adults, while no significant effects were observed for interviewer-rated or comparative health—an important null finding indicating specificity to subjective perceptions. Service-specific analyses revealed that unmet needs in personal daily care and psychological support most strongly eroded SWB, while unmet needs in social/recreational activities and psychological support uniquely shaped health status.

**Conclusions:**

Unmet community care needs negatively affect health status and SWB, but their impact varies by service types and family structure. Childlessness appears to buffer against these adverse effects, reflecting adaptive coping and institutional support. Targeted expansion of psychological support, personal daily care, and social and recreational programs, together with culturally sensitive approaches that align with informal support norms, is essential to promote aging well in China.

**Supplementary Information:**

The online version contains supplementary material available at 10.1186/s12877-025-06952-z.

## Introduction

Population aging has become a critical challenge in China. By 2024, 310 million people were aged 60 or above—22% of the total population—and this figure is projected to reach 481 million by 2050 [37, 44]. The growing demand for long-term care has strained traditional family-based care systems. Declining fertility, driven largely by the one-child policy, and rapid urbanization have reduced the availability of family caregivers [41]. As a result, formal care services are increasingly central to eldercare provision.

Despite major policy initiatives such as the Starlight Project and the “9073” eldercare framework, formal care coverage remains limited. Only 0.74% of older adults reside in long-term care facilities, far below the estimated 14% who express a preference for institutional care [36, 45]. Community care services—professional health and support services designed to promote independence among older adults living in the community—have thus emerged as a critical bridge between family-based and institutional care [8]. However, service expansion has not kept pace with rising demand, resulting in persistent unmet community care needs [31, 60, 61]. Prior studies show that unmet needs are more prevalent among rural and low-income older adults [21, 49, 57] and are shaped by factors such as marital status, education, and service accessibility [3, 5, 35]. Unmet needs have been linked to reduced life satisfaction, increased emergency visits, and poorer preventive care [24, 33, 45]. However, most studies focus on either health status or a single dimension of SWB—life satisfaction. Few examine emotional well-being—positive and negative affect—or integrated multiple health and well-being outcomes within a unified framework. Moreover, potential moderators such as urban–rural residence, disability status, and childlessness and service-specific differences remain underexplored.

**Fig. 1:**
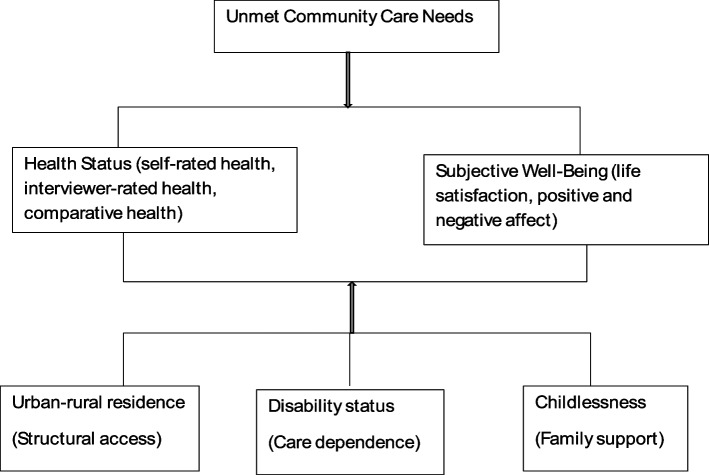
Conceptual model of the study

Building on the stress process theory [38], the conceptual model posits that unmet community care needs act as a service-related stressor that limits access to formal care resources, reduces autonomy, and increases psychological burden. Over time, these experiences may erode both health status and SWB. The model further proposes that the strength of these associations is moderated by contextual and individual factors—particularly urban–rural residence, disability status, and childlessness—which influence older adults’ capacity to compensate for unmet needs through structural access, functional independence, or family support (Fig. 1).

To address these gaps, the present study extends prior work by jointly evaluating the effects of unmet community care needs on multiple dimensions of health status and SWB—life satisfaction, positive and negative affect. It further examines whether these associations vary by urban–rural residence, service types, childlessness, and disability status. Methodologically, it advances the literature by applying two-way (person and time) fixed-effects models, strengthening causal inference by controlling for unobserved individual characteristics and period-specific influences.

## Methodology

### Data

Data were drawn from the Chinese Longitudinal Healthy Longevity Survey (CLHLS), a nationally representative panel study of older adults aged 65 and older in China. The CLHLS collects detailed information on the health status and quality of life of older adults. The analytic sample included 8,301 unique respondents aged 65 and older at baseline (2008). Owing to the panel design, respondents contributed repeated observations across subsequent waves (2012, 2014, and 2018). This resulted in a total of 24,145 person-wave observations.

Sample attrition due to mortality and non-response is reported in Supplementary Table S10, which presents continuity across survey waves. The CLHLS employed a multistage stratified cluster sampling across 22 provinces, covering 85.3% of China’s population. Sampling weights were applied to ensure national representativeness. Data were collected via face-to-face interviews and physical examinations. Respondents who participated in at least two waves were retained; after excluding singletons and missing values, the final sample included 8,301 individuals.

All CLHLS procedures involving human subjects were approved by the Duke University Health System’s Institutional Review Board and the Biomedical Ethics Committee of Peking University (Approval Number: IRB00001052-13074). Since the current study was a secondary data analysis using publicly available anonymous datasets, further ethics approval (IRB) was not required.

### Variables

#### Dependent variables

Health status was measured using three indicators: self-rated health, interviewer-rated health, and comparative health. Self-rated health was assessed by “How do you rate your health at present?” (1 = very good to 5 = very bad ). Responses were reverse-coded so that higher scores indicated better health, a measure commonly used in studies of older adults in China [32, 51]. Interviewer-rated health provided an external assessment and was recoded into a binary indicator (1 = good health, 0 = poor health). Comparative health captured perceived changes in health over the past year and was recoded as “worse” (0), “better” (1), or “no change” (2) for clarity.

SWB was measured through three components—life satisfaction, positive affect, and negative affect— to capture its complexity [11, 55]. life satisfaction was assessed by “How do you rate your life at present?” (1 = very bad to 5 = very good). Positive affect reflected emotional optimism and happiness, measured by four items (see Supplementary Appendix A1), summed into an index (range: 4–20, higher values indicate greater well-being). Negative affect captured emotional distress (e.g., fear, loneliness, and perceived uselessness) and was measured using three items summed into an index, with higher scores reflecting more negative feelings (see Supplementary Appendix A1).

#### Independent variables

Unmet community care needs were the main independent variable. It is defined as discrepancy between the community care services that older adults expect and those actually accessed [15, 28, 29, 48]. Service availability was evaluated for eight types of community care services: personal daily care, home visits, psychological consulting, daily shopping, social and recreation activities, legal aid, health education, and neighborhood relations. The latter referred specifically to formal community assistance to resolve family or neighborhood disputes (e.g., mediation, arbitration), rather than activities aimed at fostering social relationships. Respondents indicated whether each service was available (“yes”/“no”).

Service need was measured based on respondents' expectations of what community care services they believe should be provided by their community (the same eight services). Respondents indicated “Yes” or “No” for each service to express their need for that service (yes = 1, no = 0).

Unmet needs were established by the response to these two questions. A value of “1” was assigned if the respondent reported needing a service that was not available, indicating an unmet need. A value of “0” was assigned if the service was either available when needed or not needed at all, representing “no unmet need”. This conditional approach aligned with standard measures in the literature [29, 31]. The total unmet needs were a continuous composite measure that summarized the extent to which the needs of community care services were not being met. It was created by summing the individual indicators of unmet needs for each type of community care service. The value of the variable can range from 0 to 8, with a higher score indicated a greater level of unmet community care needs [1, 42, 45].

#### Control variables

Older adults’ health status and SWB were associated with multifaceted factors, such as social support, functional ability, socioeconomics, and health conditions. Referring to previous literature, this research adopted various demographic, socioeconomic, and health condition variables as control variables [33, 51, 53, 59]. Demographic variables included: age (young-old, middle-old, oldest-old), marital status, gender, ethnicity, urban–rural residence, living arrangement, childlessness, and family size. Childlessness was defined as having no surviving biological or adopted children (1 = childless, 0 = having children). Children-in-law were not counted as children, as they did not necessarily provide direct emotional or instrumental support.

Socioeconomic variables included: health insurance, education level, household income, and financial sufficiency. And health-related variables included: functional ability (ADLs), smoking, alcohol consumption, regular exercise, cognitive impairment and chronic diseases status. Functional ability was coded as “1” if respondents reported having one or more difficulties with these activities (bathing, dressing, eating, getting in/out of bed, or toileting). Chronic diseases status was coded as “1” if respondents suffered from any of a specified list of ten chronic diseases. Cognitive functioning was assessed using the Chinese Mini-Mental State Examination (MMSE) and defined as a score of 24 or below [17, 46].

#### Analytical strategy

Missing data were addressed using multiple imputations by chained equations (MICE) with 10 imputations [40]. All variables with missing values (> 0%) (e.g., household income, family size, and positive affect) were imputed using predictive mean matching for continuous and logistic models for categorical variables. All imputations incorporated demographic, socioeconomic, and health covariates to preserve panel structure and within-individual variation.

Two-way fixed-effects models with both individual and time fixed effects were employed to assess within-individual changes in unmet community care needs and their associations with health outcomes over time. This approach controls for unobserved, time-invariant individual characteristics and period-level shocks, reducing bias from omitted variables stable over time or across persons [2, 47]. Moreover, interaction terms were included to test whether the effects of unmet community care needs varied by urban–rural residence, disability status, and childlessness. Specifically, each moderator was examined in separate models to avoid overfitting and to facilitate interpretation.

Robustness was assessed through multiple checks: (1) comparing one-way (person fixed effects only) and two-way fixed-effects specifications; (2) testing nonlinearity by adding a quadratic term for unmet needs; and (3) examining whether cognitive impairment influenced reporting of unmet needs or health outcomes. All analyses were conducted using STATA version 17.

## Results

### Demographic characteristics of study participants

Table 1 summarizes the characteristics of the analytic sample. The age distribution consisted of youngest-old (54.27%), middle-old (38.64%), and oldest-old (7.09%). Women accounted for 53.25% of respondents. Most participants lived with household members (83.80%), resided in rural areas (56.87%), were married (59.12%), and identified as Han ethnicity (93.85%). A small proportion of respondents were childless (1.72%).

**Table 1:** Descriptive statistics of variables (Weighted)

Variable		Number	Percentage/Mean (SD)
Age	Youngest-Old	12,763	54.27%
	Middle-Old	9,089	38.64%
	Oldest-Old	1,668	7.09%
Sex	Male	10,996	46.75%
	Female	12,525	53.25%
Marital status	Married	13,710	59.12%
	Single	9,481	40.88%
Education level	(mean, SD)		3.27 (3.85)
Function disability (ADLs)	Yes	1,787	7.78%
	No	21,168	92.22%
Family size	(Mean, SD)		2.35 (1.71)
Chronic disease	Yes	12,893	59.21%
	No	8,883	40.79%
Residence	Urban	10,144	43.13%
	Rural	13,377	56.87%
Ethnicity	Han	20,051	93.85%
	Others	1,313	6.15%
Living Arrangements	Alone	3,681	16.20%
	With Household Members	19,040	83.80%
Household income (RMB) in 1/1000	(Mean, SD)		19.02 (19.32)
Childless	Yes	366	1.72%
	No	20,942	98.28%
Health insurance	Yes	20,356	93.65%
	No	1,381	6.35%
Financial sufficiency	Yes	18,154	78.17%
	No	5,070	21.83%

The average family size was 2.35, and the mean household income was 19,020 RMB. A significant majority of participants had health insurance (93.65%) and reported financial sufficiency (78.17%). Regarding health conditions, 59.21% reported at least one chronic disease, and 7.78% reported one or more difficulties in activities of daily living (ADLs).

### National trends in community care provision and demand

**Fig. 2:**
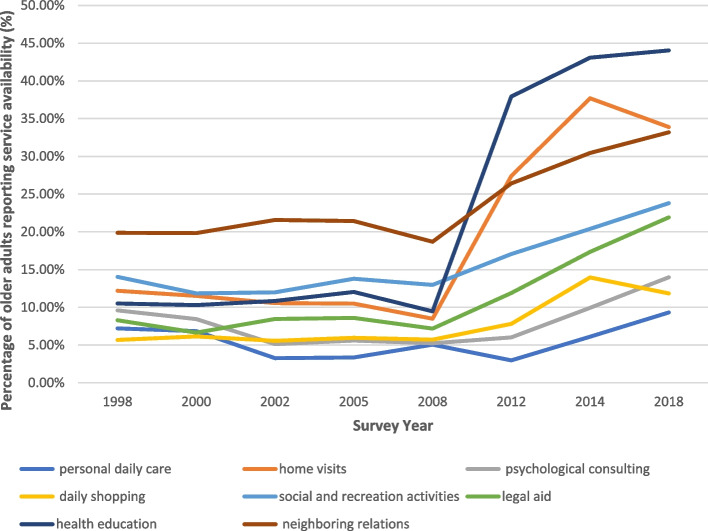
Provision of Community Care Services in China. 1998–2018. Note: Values represent the percentage of older adults reporting that each service was available. All numbers are weighted. N (1998) = 9,093; N (2000) = 11,199; N (2002) = 16,020; N (2005) = 15,613; N (2008) = 16,563; N (2012) = 9,679; N (2014) = 7,107; N (2018) = 15,779. Source: Data derived from the CLHLS 1998–2018

**Fig. 3:**
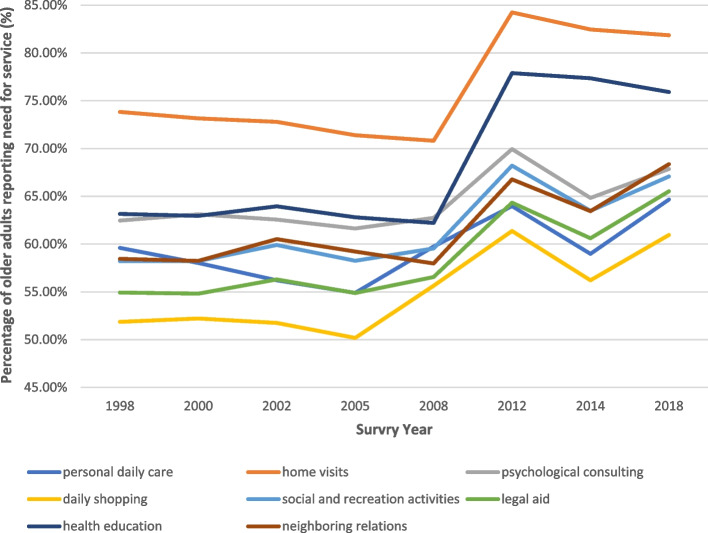
Demand of Community Care Services in China, 1998–2018. Note: Values represent the percentage of older adults reporting that each service was needed. All numbers are weighted. N (1998) = 9,093; N (2000) = 11,199; N (2002) = 16,020; N (2005) = 15,613; N (2008) = 16,563; N (2012) = 9,679; N (2014) = 7,107; N (2018) = 15,779. Source: Data derived from the CLHLS 1998-2018

Figures 2 and 3 illustrate national trends in provision and demand for community care services between 1998 and 2018. The availability of community care services in China increased substantially between 1998 and 2018, particularly after 2008, coinciding with national initiatives such as the Starlight Program and the “9073” eldercare framework. Growth was most pronounced for personal daily care, home visits, and neighborhood relations services. However, demand consistently outpaced provision. For instance, although 82.16% of older adults reported a need for home visits, only 33.89% reported that such services were available in their communities. This widening gap underscores ongoing structural constraints in community care delivery and the prevalence of unmet community care needs among older adults in China.

### Fixed-effects model results

Table 2 presented fixed-effect estimates of the relationship between unmet community care needs and health status. Unmet needs were negatively associated with self-rated health (β = − 0.005, *p* < 0.05), indicating that within individuals, increases in unmet needs were associated with poorer self-rated health over time. In contrast, unmet community care needs had no significant effect on interviewer-rated or comparative health. These null results suggest that the effects of unmet needs were reflected primarily in older adults’ subjective health perceptions rather than in externally assessed or comparative health measures.

**Table 2:** Associations between unmet community care needs and health status

Variables	Model 1 Self-rated health	Model 2 Interviewer-rated health	Model 3 Comparative health
Unmet needs	−0.005**	−0.001	−0.001
Age	0.032	0.050***	−0.008
Age^2^	−0.000	−0.000***	0.000
Family size	0.004	0.002	0.002
Rural (ref: Urban)	0.000	0.001	0.007
Married (ref: Single)	0.017	0.009	−0.017
Living alone (ref: living with family members)	0.035	0.021	−0.018
Financial sufficiency	0.333***	0.082***	0.227***
Regular exercise	0.172***	0.056***	0.083***
Alcohol consumption	0.077**	0.017	0.001
Smoking	0.077**	0.036***	0.022
Chronic diseases (ref: Without chronic diseases)	−0.214***	−0.063***	−0.188***
Health insurance	−0.105***	−0.078***	0.008
Household income	0.001	0.001***	−0.000
Childless (ref: With Children)	−0.008	−0.017	0.134
Functional ability (ADLs)	−0.291***	−0.215***	−0.236***
Year (ref: 2008)			
2012	−0.151***	−0.033**	−0.110***
2014	−0.228***	−0.066***	−0.132
2018	−0.214**	−0.108***	−0.081
Observations	24,145	24,145	24,145

β: regression coefficient

Household income used in 1/1000

^**^P < 0.05, and ***p < 0.01

Table 3 presents the associations between unmet community care needs and SWB. Unmet needs were significantly associated with lower life satisfaction (β = − 0.010, *p* < 0.01) and reduced positive affect (β = −0.024, *p* < 0.01). Unmet needs were also associated with higher negative affect (β = 0.023, *p* < 0.01). Although the estimated coefficients appear small in magnitude, they represent within-individual changes that may accumulate to meaningful differences in well-being over time [19, 34].

**Table 3:** Association between unmet community care needs and SWB

Variables	Model 1 Life satisfaction	Model 2 Positive affect	Model 3 Negative affect
Unmet needs	−0.010***	−0.024***	0.023***
Age	0.024	−0.033	0.114
Age^2^	−0.000	−0.000	−0.000
Family size	0.010	−0.010	−0.040
Rural (ref: Urban)	−0.027	−0.128**	0.080
Health status	0.345***	0.732***	−0.586***
Married (ref: Single)	0.004	0.308***	−0.490***
Living alone (ref: living with family members)	−0.049	0.413***	0.222**
Financial sufficiency	0.296***	0.487***	−0.656***
Chronic diseases (ref: Without chronic diseases)	0.007	0.007	0.107
Health insurance	0.001	−0.090	0.023
Household income	0.003***	0.004***	−0.001
Functional ability (ADLs)	−0.023	−0.552***	0.334***
Childless (ref: With Children)	0.025	0.115	0.233
Year (ref: 2008)			
2012	0.079***	0.840***	−0.111
2014	0.165***	0.658***	−0.142
2018	0.226***	0.548	−0.315
Observations	24,145	24,145	24,145

β: regression coefficient

Household income used in 1/1000

^**^P < 0.05, and ***p < 0.01

### Associations between service-specific unmet needs, health status, and SWB

Table 4 breaks down unmet community care needs by service type. Unmet needs in psychological support had the strongest negative association with self-rated health (β = –0.071, *p* < 0.05). For interviewer-rated health, only unmet needs in social and recreational activities showed a significant negative impact (β = −0.022, *P* < 0.05). In contrast, no service-specific unmet needs were significantly associated with comparative health.

**Table 4:** Associations between unmet community care needs and health status

Unmet needs	Model 1 Self-rated health	Model 2 Interviewer-rated health	Model 3 Comparative health
Home Visits	0.015 (0.020)	0.009 (0.008)	−0.003 (0.022)
Personal Daily Care	−0.035 (0.045)	0.004 (0.017)	0.018 (0.049)
Psychological Support	−0.071** (0.034)	−0.011 (0.013)	−0.061 (0.038)
Daily Shopping	−0.019 (0.035)	−0.004 (0.014)	−0.021 (0.039)
Social and Recreational Activities	−0.030 (0.026)	−0.022** (0.010)	−0.017 (0.029)
Legal Aid	0.001 (0.030)	0.004 (0.011)	−0.018 (0.034)
Health Education	0.014 (0.021)	−0.004 (0.008)	0.012 (0.024)
Neighborhood Relations	−0.023 (0.022)	−0.012 (0.008)	0.015 (0.025)

β: regression coefficient. The numbers in parentheses are the standard errors. **p < 0.05, ***p < 0.01. Controls include age, age^2^, family size, urban–rural residence, marital status, living arrangement, financial sufficiency, regular exercise, alcohol consumption, smoking, health insurance, household income, childlessness, chronic diseases, year FE

Table 5 presents services-specific unmet needs associations with SWB. Specifically, unmet needs in all services—except daily shopping and psychological support— were significantly associated with lower life satisfaction. Unmet needs in personal daily care exerted the strongest negative influence on life satisfaction (β = −0.099, *p* < 0.01). For positive affect, unmet needs in psychological support emerged as the most significant predictor (β = –0.203, *p* < 0.05). Conversely, unmet needs in neighborhood relations were unexpectedly associated with lower negative affect (β = –0.144, *p* < 0.05).

**Table 5:** Relationship between unmet community care needs and SWB

Unmet needs	Model 1 Life satisfaction	Model 2 Positive affect	Model 3 Negative affect
Home Visits	−0.047*** (0.017)	−0.021 (0.055)	−0.036 (0.053)
Personal Daily Care	−0.099*** (0.037)	−0.071 (0.119)	0.016 (0.110)
Daily Shopping	−0.016 (0.030)	−0.095 (0.096)	0.047 (0.088)
Psychological Support	−0.052 (0.028)	−0.203** (0.089)	−0.010 (0.085)
Social and Recreational Activities	−0.067*** (0.022)	−0.068 (0.069)	−0.034 (0.069)
Legal Aid	−0.057** (0.025)	0.077 (0.084)	0.035 (0.077)
Health Education	−0.040** (0.017)	−0.058 (0.057)	−0.076 (0.057)
Neighborhood Relations	−0.055*** (0.018)	−0.081 (0.060)	−0.144** (0.055)

β: regression coefficient. The numbers in parentheses are the standard errors. **p < 0.05, ***p < 0.01. Controls include age, age^2^, family size, urban–rural residence, self-rated health, marital status, living arrangement, financial sufficiency, chronic diseases, health insurance, household income, Functional ability (ADLs), childlessness, year FE

### Interaction effect

Table 6 reported the moderating role of childlessness in the associations between unmet community care needs and health outcomes. Unmet community care needs were negatively associated with self-rated health (β = −0.007, *p* < 0.01). The interaction term between unmet needs and childlessness was positive and statistically significant (β = 0.033, *p* < 0.05), indicating that the adverse impact of unmet needs on self-rated health was weaker among childless older adults than among those with children.

**Table 6:** Effect of unmet community care needs on health status by childlessness

Variables	Model 1 Self-rated health	Model 2 Interviewer-rated health	Model 3 Comparative health
Unmet needs	−0.007***	−0.001	−0.001
Childless (ref: With children)	−0.124	−0.025	0.085
Unmet needs × Childless	0.033**	0.001	0.012
Age	−0.005	0.050***	−0.007
Age^2^	0.000	−0.000***	0.000
Family size	0.005	0.002	0.000
Rural (ref: Urban)	0.001	0.000	0.005
Married (ref: Single)	0.004	0.009	−0.015
Regular exercise	0.143***	0.056***	0.084***
Alcohol consumption	0.058**	0.017	−0.000
Smoking	0.069**	0.035***	0.021
Financial sufficiency	0.310***	0.083***	0.226***
Chronic diseases (ref: Without chronic diseases)	−0.212***	−0.063***	−0.185***
Living alone (ref: living with family members)	0.020	0.020	−0.025
Functional ability (ADLs)	−0.286***	−0.216***	−0.228***
Health insurance	−0.058**	−0.077***	0.020
Household income	0.001***	0.001***	−0.000
Year (ref: 2008)			
2012	−0.101***	−0.033**	−0.114***
2014	−0.175***	− 0.067***	− 0.136**
2018	− 0.144	− 0.110***	− 0.092
Observations	24,145	24,145	24,145

β: regression coefficient

Household income used in 1/1000

^**^P < 0.05, and ***p < 0.01

The moderating effect of childlessness remained robust across multiple sensitivity checks, including alternative categorical operationalizations of unmet needs (β = –0.017, *p* < 0.01) and models using person fixed effects only (β = –0.007, *p* < 0.01) (Supplementary Tables S8). These results suggest that the buffering role of childlessness is not driven by measurement bias or model specification.

However, childlessness did not moderate interviewer-rated or comparative health outcomes, indicating that moderation was specific to self-rated health.

Table 7 reports interaction models testing urban–rural residence and disability status as moderators. None of the interaction terms were statistically significant (all *p* > 0.05), indicating consistent impacts across these subpopulations.

**Table 7:** Moderating effect of disability and residence on health status and SWB

Variables	Model 1 Self- rated health	Model 2 Self-rated health	Model 3 Life satisfaction	Model 4 Life satisfaction
Unmet Needs	−0.006**	−0.006***	−0.014***	−0.010***
Disabled (ref: non-disabled)	−0.282***	-	-	−0.026
Disabled × Unmet needs	−0.001	-	-	0.001
Rural (ref: Urban)	-	−0.001	−0.053**	-
Rural × Unmet needs	-	0.0004	0.007	-
Observations	24,145	24,145	24,145	24,145

Note: the covariates are hidden from the table

β: regression coefficient

^**^P < 0.05, and ***p < 0.01

### Sensitivity analysis

Findings remained robust across alternative model specifications and sensitivity analyses (Supplementary Table S2-S7). To assess potential nonlinearity, squared terms of unmet needs were included. The squared term was statistically significant for positive affect, negative affect, and interviewer-rated health, but not for life satisfaction, self-rated health, and comparative health (Supplementary Table S6). For comparability, we presented the linear specification in the main text and provided robustness checks with quadratic terms in the Supplementary materials (Supplementary Table S7).

Additional analyses examined whether cognitive impairment moderated the associations between unmet community care needs and well-being. Results showed no statistically significant interaction effects (Supplementary Tables S2–S4). However, stratified analyses revealed stronger negative associations among cognitively impaired older adults (β = –0.054) than among cognitively intact peers (β = –0.008; Supplementary Table S4).

## Discussion

Consistent with prior research [4, 14, 24, 30, 33, 45], higher levels of unmet community care needs were associated with poorer self-rated health and lower SWB, including reduced life satisfaction, diminished positive affect, and increased negative affect. Together, these results indicate that unmet community care needs remain a critical determinant of older adults’ well-being despite national initiatives such as the Starlight Project and the “9073” eldercare framework, likely by heightening psychological stress and reducing autonomy [25, 35, 43]. While prior studies emphasized psychosocial resilience as a protective factor [13, 45], this study extended the literature by identifying childlessness as a structural moderator that mitigated the negative association between unmet community care needs and health outcomes.

A key contribution of this study lies in identifying childlessness as a potential adaptive moderator. The negative association between unmet community care needs and self-rated health was weaker among childless older adults. This finding contrasted with studies portraying childless older adults as more vulnerable due to limited family support [18, 22, 58] and instead aligns with frameworks of long-term adaptation and resilience. In the Chinese context—where filial piety remains a dominant cultural norm—older adults expect direct support from their children. Older adults with children may experience greater disappointment and distress when expected support is not realized [54]. By contrast, childless older adults, having long adapted to self-reliance and lower expectations of family-based care, may have perceived unmet community care needs as less of a social deficit [7, 23]. From a life-course perspective, Cumulative Advantage/Disadvantage Theory suggests that early-life disadvantages such as childlessness may cultivate compensatory resilience over time [16, 39]. Childless older adults may have developed stronger coping mechanisms, greater self-efficacy, and higher engagement with community-based care services to offset the absence of family support [9, 26, 56]. These adaptive capacities, reinforced by institutional support, may buffer them against unmet needs.

Beyond family context, the study’s service-specific analysis revealed meaningful variation across types of community care services. Unmet needs in personal daily care and psychological support were most detrimental to SWB, while unmet needs in social and recreational activities and neighborhood relations had distinct effects on health status. These results underscored that not all unmet needs were equally consequential, and that policymakers should prioritize the services most closely tied to well-being. The application of two-way fixed-effects models, a key methodological contribution, strengthened the robustness of these findings by accounting for unobserved individual heterogeneity.

One finding diverged from prior literature [33, 45]: unmet needs in neighborhood relations were associated with lower negative affect. This unexpected relationship may reflect cultural and psychological factors unique to the Chinese context. Neighborhood relations services often serve as formal mechanisms for conflict mediation. However, older adults may prefer informal dispute resolution through family elders or trusted neighbors to avoid public confrontation—a strategy consistent with Confucian values of harmony and indirect communication [6, 10, 27]. In this context, reporting unmet needs may reflect a cultural preference for privacy rather than a genuine service deficit. Measurement bias and suppression effects may also be at play: individuals living in cohesive, low-conflict neighborhoods may report limited availability of neighborhood relations services because such services are unnecessary, while simultaneously experiencing lower psychological distress due to strong informal social ties [12, 52]. These dynamics could suppress the expected positive association between unmet neighborhood relations needs and negative affect.

In contrast, urban–rural residence and disability status did not significantly moderate the associations between unmet community care needs and health status or SWB. The null urban–rural findings were unexpected given China’s health disparities in service access [50]. However, persistent system-wide service shortages across both areas may have reduced differential effects [45]. Similarly, the absence of moderation by disability status suggested that unmet community care needs undermined well-being universally, rather than disproportionately affecting specific functional groups [20, 22].

Stratified analyses by cognitive status revealed that cognitively impaired older adults experienced stronger negative associations between unmet needs and well-being compared with cognitively intact counterparts (Supplementary Table S4). Although the interaction term was not statistically significant, this pattern suggests heightened vulnerability among cognitively impaired individuals and highlights the importance of targeted interventions for this subpopulation.

These findings have important implications for China’s ongoing efforts to build an equitable, sustainable community care system. First, the consistent association between unmet needs and poorer health and SWB highlights the urgency of expanding service availability and accessibility, particularly personal daily care, psychological support, and social/recreational activities. Second, the moderating role of childlessness suggests that policy approaches should move beyond deficit-based assumptions and recognize adaptive resilience among childless older adults, leveraging strengths through peer-support groups, volunteer engagement, and self-help programs. Finally, the results regarding neighborhood relations underscore the importance of culturally sensitive interventions that complement informal support structures and respect norms of relational harmony.

## Limitations

There are limitations to acknowledge. First, unmet community care needs were measured through self-reports, which may not fully align with actual service utilization. Older adults may underreport needs due to stigma or limited awareness, particularly for psychosocial services. Future research could improve reliability by cross-validating self-reported measures with community-level records or proxy reports, especially for cognitively impaired respondents. Second, the findings are specific to China’s aging population and may not be generalizable to other countries. This limits generalizability to other contexts, but it can also be viewed as a strength. At the same time, China represents a critical case of rapid population aging and community care reform, offering insights relevant to other aging societies. Finally, the study does not account for differences in local policies or the design of community care services, which can vary significantly across provinces or urban–rural settings. Such variations could affect access, quality, and perceptions of unmet needs in community care services. Future research could examine how such differences shape unmet needs and well-being.

## Conclusion

This study provides robust longitudinal evidence that unmet community care needs undermine both health status and SWB among older adults in China. By identifying childlessness as a moderating factor, the findings highlight how family structure and lifelong adaptation shape individuals’ responses to care deprivation. Contrary to assumptions that childless older adults are uniformly more vulnerable, the findings suggest that adaptive coping and institutional support may buffer their well-being, offering a new perspective on resilience in later life.

The service-specific analysis further demonstrates that unmet needs are not all equally consequential: unmet needs in personal daily care and psychological support most strongly predict declines in SWB, while unmet needs related to neighborhood relations and social/recreational activities have unique implications on health status. The null moderating effects for urban–rural residence and disability status suggest that unmet community care needs represent a broad, system-wide challenge rather than one confined to specific subpopulations.

These insights emphasize the urgency of prioritizing high-impact community services while designing inclusive, family-sensitive programs that recognize the diversity of older adults’ caregiving contexts. Addressing unmet community care needs requires not only expanding service availability but also aligning interventions with cultural norms of relational harmony and individual adaptive capacity. Future research should continue to explore cross-cultural generalizability and assess how specific policy reforms can reduce unmet community care needs to promote an equitable, sustainable community care system.

## Supplementary Information

Supplementary Material 1.

## Data Availability

The datasets generated and/or analyzed during the current study are available in the Zenodo repository: [https://doi.org/10.5281/zenodo.17988307](https://doi.org/10.5281/zenodo.17988307).

## Abbreviations

SWB: Subjective Well-being
CLHLS: Chinese Longitudinal Healthy Longevity Survey
ADLs: Activities of Daily Living
MMSE: Mini-Mental State Examination
