# Supplementary material for: Unmet community care needs and older adults’ well-being: the moderating role of childlessness in China
Source: BMC Geriatr. 2026 Feb 4;26:309. doi: 10.1186/s12877-025-06952-z (PMC12964614; doi:10.1186/s12877-025-06952-z)
Supplement: Supplementary file 1 — Supplementary Material 1. [file 12877_2025_6952_MOESM1_ESM.docx]

**Supplementary Materials**

Supplementary Appendix A1 Measures of Subjective Well-Being

Positive Affect (4 items, summed index: 4–20, higher = greater well-being)

Responses: 1 = always, 2 = often, 3 = sometimes, 4 = seldom, 5 = never; 8 = not able to answer (reverse-scored).

1. Do you always maintain an optimistic outlook?
2. Do you consider yourself as happy now as when you were younger?
3. Do you feel capable of making your own decisions concerning personal affairs?
4. Do you take satisfaction in keeping your surroundings tidy?

Negative Affect (3 items, summed index: 3–15, higher = more negative feelings)

Responses: 1 = always, 2 = often, 3 = sometimes, 4 = seldom, 5 = never; 8 = not able to answer.

1. Do you often feel fearful or anxious?
2. Do you often feel lonely and isolated?
3. Do you believe that aging has made you feel more useless?

Supplementary Table S1 Descriptive Statistics by Cognitive Status

Caption: Sample means and SDs for key variables, stratified by cognitive impairment (MMSE ≤24 = impaired; >24 = intact). Impaired group shows slightly higher unmet needs (mean=4.04 vs. 3.94) and lower SWB/health status, justifying sensitivity checks for bias.

| Variable | Cognitively Intact |  | Cognitively Impaired |  | Total |  |
| --- | --- | --- | --- | --- | --- | --- |
|  | Mean | SD | Mean | SD | Mean | SD |
| Life Satisfaction | 3.75 | 0.83 | 3.65 | 0.81 | 3.74 | 0.83 |
| Unmet Needs | 3.94 | 3.13 | 4.04 | 3.17 | 3.95 | 3.13 |
| Self-Rated Health | 3.41 | 0.93 | 3.31 | 0.91 | 3.41 | 0.93 |
| Age | 84.51 | 10.49 | 87.93 | 9.76 | 84.69 | 10.48 |
| Family Size | 2.49 | 1.70 | 2.56 | 1.64 | 2.49 | 1.70 |
| Income | 20,618.66 | 20,259.05 | 19,158.75 | 19,637.58 | 20,541.07 | 20,228.69 |

Supplementary Table S2 Interaction Model Results for Cognitive Impairment

Caption: Cognitive impairment was defined using validated screening criteria from the CLHLS cognitive module (MMSE-based classification; 1 = impaired, 0 = intact). Full-sample fixed-effects interactions between unmet needs levels (1–8) and cognitive impairment (ref: intact) on life satisfaction. Model 1: Unadjusted levels; Model 2: With covariates. Most interactions non-significant (p > 0.05), but Level 7 shows moderation (β = -0.24**, p < 0.05 in Model 1), suggesting nuance at higher unmet counts. No systematic bias overall.

| Variables | Model 1 | Model 2 |
| --- | --- | --- |
| Unmet Needs (Level 1) | 0.036 | 0.018 |
| Unmet Needs (Level 2) | 0.022 | 0.002 |
| Unmet Needs (Level 3) | 0.007 | 0.001 |
| Unmet Needs (Level 4) | -0.003 | 0.000 |
| Unmet Needs (Level5) | -0.019 | -0.011 |
| Unmet Needs (Level 6) | -0.054 | -0.049 |
| Unmet Needs (Level 7) | -0.039 | -0.034 |
| Unmet Needs (Level 8) | -0.105*** | -0.063*** |
| Cognitive Impairment | -0.041 | -0.007 |
| Unmet Needs × Impairment (1) | 0.044 | 0.064 |
| Unmet Needs × Impairment (2) | 0.063 | 0.054 |
| Unmet Needs × Impairment (3) | -0.021 | -0.004 |
| Unmet Needs × Impairment (4) | -0.026 | 0.001 |
| Unmet Needs × Impairment (5) | 0.052 | 0.016 |
| Unmet Needs × Impairment (6) | -0.120 | -0.172 |
| Unmet Needs × Impairment (7) | -0.226 | -0.142 |
| Unmet Needs × Impairment (8) | -0.134 | -0.099 |
| Age |  | 0.028 |
| Age^2 |  | -0.000 |
| Family size |  | 0.010 |
| Rural (ref: Urban) |  | -0.023 |
| Health status |  | 0.345*** |
| Married (ref: Single) |  | 0.008 |
| Living alone (ref: living with family members) |  | -0.053** |
| Financial sufficiency |  | 0.292*** |
| Chronic diseases (ref: Without chronic diseases) |  | 0.009 |
| Health Insurance |  | 0.000 |
| Household Income |  | 0.003*** |
| Function ability (ADL) |  | -0.020 |
| Childless (ref: With Children) |  | 0.027 |
| Year 2012 |  | 0.075** |
| Year 2014 |  | 0.161*** |
| Year 2018 |  | 0.223** |
| Constant | 3.77*** | 1.08 |
| Observations | 24,145 | 24,145 |
| β: regression coefficient |  |  |
| Household income used in 1/1000.  **P< 0.05, and *** p < 0.01 |  |  |

Supplementary Table S3 Stratified Analysis Results by Cognitive Status

Caption: Fixed-effects coefficients (β) and SEs for life satisfaction models, stratified by cognitive impairment (impaired subsample: n=1,251; intact: n=22,894). Unmet needs effect stronger in impaired group (β = -0.06, SE = 0.02; p < 0.05 implied by SE), highlighting vulnerability despite non-moderation. Covariates consistent across strata.

| **Variable** | **Cognitively Impaired** |  | **Cognitively Intact** |  |
| --- | --- | --- | --- | --- |
|  | **β** | **SE** | **β** | **SE** |
| **Unmet Needs** | **-0.054** | **0.025** | **-0.008***** | **0.002** |
| **Age** | **0.075** | **0.264** | **0.030** | **0.018** |
| Age^2 | **-0.001** | **0.001** | **-0.000** | **0.000** |
| **Family Size** | **-0.075** | **0.075** | **0.010** | **0.005** |
| Rural (ref: Urban) | **-0.076** | **0.213** | **-0.021** | **0.016** |
| Health status | **0.234** | **0.093** | **0.349***** | **0.007** |
| Married (ref: Single) | **-0.792** | **0.427** | **0.016** | **0.026** |
| Living alone (ref: living with family members) | **-0.558** | **0.283** | **-0.045** | **0.026** |
| Financial sufficiency | **0.128** | **0.207** | **0.297***** | **0.016** |
| Chronic diseases (ref: Without chronic diseases) | **-0.064** | **0.186** | **0.009** | **0.014** |
| Health Insurance | **-0.050** | **0.179** | **-0.001** | **0.018** |
| Household Income | **0.004** | **0.005** | **0.003***** | **0.000** |
| **ADL Function** | **-0.366** | **0.198** | **-0.016** | **0.020** |
| **Year FE** | **Yes** |  | **Yes** |  |
| **Observations** | **1,251** |  | **22,894** |  |

| β: regression coefficient |
| --- |
| Household income used in 1/1000.  **P< 0.05, and *** p < 0.01 |

Supplementary Table S4 Robustness Check: Person Fixed Effects vs. Two-Way Fixed Effects

| Outcome Variable | Model Type | Coefficient of Unmet Needs (β) | SE | p-value |
| --- | --- | --- | --- | --- |
| Self-Rated Health | Person FE | -0.0073 | 0.0023 | 0.001 |
|  | Two-Way FE | -0.0074 | 0.0023 | 0.001 |
| Life Satisfaction | Person FE | -0.0132 | 0.0021 | <0.001 |
|  | Two-Way FE | -0.0131 | 0.0021 | <0.001 |
| Positive Affect | Person FE | -0.0340 | 0.0065 | <0.001 |
|  | Two-Way FE | -0.0338 | 0.0065 | <0.001 |
| Negative Affect | Person FE | 0.0307 | 0.0060 | <0.001 |
|  | Two-Way FE | 0.0308 | 0.0060 | <0.001 |
| Interviewer-Rated Health | Person FE | -0.0012 | 0.0008 | 0.138 |
|  | Two-Way FE | -0.0013 | 0.0008 | 0.116 |
| Comparative Health | Person FE | -0.0010 | 0.0025 | 0.683 |
|  | Two-Way FE | -0.0008 | 0.0025 | 0.760 |

Supplementary Table S5 Comparison of Fixed-Effects Estimates Using Continuous and Categorical Specifications of Unmet Community Care Needs

| Outcome Variable | Continuous |  | Categorical |  |
| --- | --- | --- | --- | --- |
|  | β | SE | β | SE |
| Self-rated health | -0.007** | 0.002 | -0.015** | 0.006 |
| Interviewer-rated health | -0.001 | 0.001 | -0.002 | 0.002 |
| Comparative health | -0.001 | 0.002 | -0.002 | 0.007 |
| Life satisfaction | -0.009*** | 0.002 | -0.022*** | 0.005 |
| Positive affect | -0.023*** | 0.006 | -0.062*** | 0.016 |
| Negative affect | 0.021*** | 0.006 | 0.068*** | 0.015 |

Notes: Models estimated using two-way fixed effects (person and year) with 10 multiple imputations by chained equations (Rubin, 1987). Control variables include age, age squared, family size, urban residence, spouse status, living arrangement, financial support, chronic disease, insurance, baseline income, childless status, ADL limitation, and health behaviors (exercise, alcohol, and smoking), as well as year dummies. ***p < 0.01, **p < 0.05.

Supplementary Table S6. Testing Nonlinearity in the Association Between Unmet Community Care Needs and Health Status and SWB

| Outcome | Linear β (SE) | Quadratic β (SE) | p-value (Quadratic) |
| --- | --- | --- | --- |
| Life satisfaction | -0.010 (0.002) | -0.001 (0.001) | 0.082 |
| Positive affect | -0.025 (0.006) | 0.006 (0.003) | 0.031 |
| Negative affect | 0.024 (0.006) | -0.012 (0.003) | 0.000 |
| Self-rated health | -0.006 (0.002) | -0.001 (0.001) | 0.525 |
| Interviewer-rated health | -0.001 (0.001) | -0.001 (0.000) | 0.007 |
| Comparative health | -0.001 (0.002) | 0.001 (0.001) | 0.553 |

Table S7 Robustness Check: Nonlinear Associations Between Unmet Needs and Well-Being Outcomes

| **Outcome Variable** | **Model Type** | **Coefficient on unmet_needs** | **Coefficient on unmet_needs_sq** | **p-value** |
| --- | --- | --- | --- | --- |
| **Positive Affect** | Linear | -0.0236*** (0.0059) | – | – |
|  | Quadratic | -0.0250*** (0.0060) | 0.0062* (0.0028) | 0.031 |
| **Negative Affect** | Linear | 0.0218*** (0.0055) | – | – |
|  | Quadratic | 0.0244*** (0.0056) | -0.0116*** (0.0027) | <0.001 |
| **Interviewer-Rated Health** | Linear | -0.0010 (0.0008) | – | – |
|  | Quadratic | -0.0010 (0.0008) | -0.0011** (0.0004) | 0.007 |

As shown in Table S7, the squared term for unmet needs was statistically significant across multiple well-being outcomes, suggesting nonlinear associations. Specifically, for positive affect, the squared term indicates a weak U-shaped relationship—initial increases in unmet needs were associated with declines in positive affect, but the effect flattened at higher levels of unmet needs. In contrast, for negative affect, the negative squared term suggests an inverted U-shaped association, where moderate levels of unmet needs corresponded to slightly higher negative affect, followed by a plateau or slight decline at high levels. For interviewer-rated health, the squared term implies a steeper deterioration in health status as unmet needs accumulated, reflecting compounding negative effects at higher unmet-need levels.

Supplementary Table S8. Robustness Checks for the Moderating Role of Childlessness in the Association Between Unmet Community Care Needs and Self-Rated Health

| Variables | Model 1: Alternative Operationalization of Unmet Needs (Categorical) | Model 2: Person Fixed Effects Only |
| --- | --- | --- |
| Unmet needs | -0.017** | -0.007*** |
| Childless (ref: With children) | -0.161 | -0.121 |
| Unmet needs × Childless | 0.096** | 0.033** |
| Age | -0.004 | -0.018 |
| Age² | 0.000 | 0.000 |
| Family size | 0.005 | 0.005 |
| Rural (ref: Urban) | 0.001 | 0.008 |
| Married (ref: Single) | 0.004 | 0.001 |
| Regular exercise | 0.143*** | 0.140*** |
| Alcohol consumption | 0.058** | 0.058** |
| Smoking | 0.069** | 0.071** |
| Financial sufficiency | 0.310*** | 0.312*** |
| Chronic diseases (ref: Without chronic diseases) | -0.212*** | -0.215*** |
| Living alone (ref: living with family members) | 0.020 | 0.011 |
| Functional ability (ADL) | -0.286*** | -0.300*** |
| Health insurance | -0.057** | -0.068** |
| Household income | 0.001** | 0.001** |
| Year FE | Yes | No |
| Observations | 24,145 | 24,145 |

β: regression coefficient

Household income used in 1/1000.

Unmet needs were modeled as a categorical variable (none = 0, low = 1–2, moderate = 3–5, high = 6–8)

**P< 0.05, and *** p < 0.01

Supplementary Table S9. Definition and Measurement of Control Variables

| Variable Name | Type | Measurement & Categories | Reference Category |
| --- | --- | --- | --- |
| Age | Categorical | 1 = Youngest-old (65–74), 2 = Middle-old (75–84), 3 = Oldest-old (85+) | Youngest-old |
| Sex | Binary | 0 = Male, 1 = Female | Male |
| Marital Status | Binary | 0 = Single (widowed/divorced/never married), 1 = Married | Married |
| Education Level | Continuous | Years of schooling | N/A |
| Functional Disability (ADL) | Binary | 0 = No difficulty in activities of daily living, 1 = Any difficulty | No difficulty |
| Family Size | Continuous | Number of household members | N/A |
| Chronic Disease | Binary | 0 = No chronic conditions, 1 = At least one chronic condition | No chronic disease |
| Residency | Binary | 0 = Urban, 1 = Rural | Urban |
| Ethnicity | Binary | 0 = Han, 1 = Others | Han |
| Living Arrangements | Binary | 0 = Alone, 1 = With household members | With household members |
| Household Income | Continuous | Annual household income divided by 1,000 RMB | N/A |
| Childless | Binary | 0 = has children, 1 = no living children | Has children |
| Health Insurance | Binary | 0 = don’t have any health insurance at present , 1 = has health insurance at present | N/A |
| Financial Sufficiency | Binary | 0 = don’t have enough financial support pay your daily cost, 1 = has sufficient financial resources to cover daily expenses | N/A |

**Note:** *Childless* refers to having no living biological or adopted children. It excludes children-in-law.

Supplementary Table S10 Continuity of Participants Across Waves in the Study, 2008 to 2018

| Waves | Total Participants | Present in 2012 | Present in 2014 | Present in 2018 |
| --- | --- | --- | --- | --- |
| 2008 | 16,563 | 8,293 | 5,135 | 2,416 |
| 2012 | 9,656 | - | 5,135 | 2,416 |
| 2014 | 5,135 | - | - | 2,416 |
| 2018 | 2,416 | - | - | - |

Note: This table presents sample continuity across waves. Of the 16,563 participants interviewed in 2008, 8,293 were re-interviewed in 2012, 5,135 in 2014, and 2,416 in 2018. Attrition primarily reflects mortality and loss to follow-up, which are common in longitudinal studies of older adults. The analytic sample used in this study consisted of 8,301 unique respondents contributing to 24,145 person-wave observations across the four waves.
